# Numerical Investigation of the Infill Rate upon Mechanical Proprieties of 3D-Printed Materials

**DOI:** 10.3390/polym14102022

**Published:** 2022-05-16

**Authors:** Laszlo Racz, Mircea Cristian Dudescu

**Affiliations:** Department of Mechanical Engineering, Faculty of Automotive, Mechatronics and Mechanical Engineering, Technical University of Cluj-Napoca, 103-105 Muncii Boulevard, 400641 Cluj-Napoca, Romania; sraczlaszlo@yahoo.com

**Keywords:** fused deposition modeling, finite element analysis, tensile testing, infill rate

## Abstract

The paper proposes a novel method of numerical simulation of the fused deposition molding 3Dprinted parts. The single filaments are modeled by a script using the G-code of the 3D printer. Based on experimental evaluation of the cross-sectional geometry of a printed tensile specimen, the connection between the filaments is determined and the flattening effect of the filaments can be counted. Finite element (FE) simulations considering different element lengths were validated by experimental tests. The methodology allows, on one hand, numerical estimation of the true cross-sectional area of a specimen and correction of the experimental stress-strain curves and, on the other hand, accurate determination of the E-modulus of a printed tensile specimen with different deposition densities (20%, 40%, 60%, 80% and 100% infill rate). If the right method to connect the single filaments is established and validated for a 3D printer, the mechanical properties of the 3D specimens can be predicted without physical tensile test, only using FE method, which will allow the designers to print out the parts with variable infill rate and tunable stiffness only after the FE result are suitable for their needs, saving considerably materials and time.

## 1. Introduction

Additive manufacturing or three-dimensional printing (3D) technologies demonstrated over the last few years a great potential to produce parts from a digital model cost efficiently, without the need of additional tooling and assembly. Three-dimensional printing successfully integrates the design and manufacturing process with an efficient use of material and ability to create parts with highly complex geometries. A very popular 3D printing method for creating prototypes or functional parts out of thermoplastics is Fused Deposition Modeling (FDM) [1], a type of material extrusion additive manufacturing technique, also known as Fused Filament Fabrication (FFF). The FDM technique is able to construct physical parts out of a range of thermoplastic materials such as acrylonitrile butadiene styrene (ABS), polylactic acid (PLA), polycarbonate, polyether-ether-ketone (PEEK) or fiber reinforced thermoplastics. The FDM process consists of the deposition of thermoplastic filaments in a semi-molten state through a heated deposition nozzle onto the build platform contributing layer by layer to the constructed part. 

Despite all these advantages, parts created with FDM technique present inferior mechanical properties, due to additional porosity and anisotropy caused by the nature of the manufacturing process. In this regard, both porosity and mechanical anisotropy strongly depend on the printing parameters [2,3,4,5,6,7,8,9]. Therefore, the influence of printing parameters may be used to customize the mechanical properties of the printed components. FDM technique has the potential to produce parts with locally controlled properties [10,11,12,13] by changing the deposition density (infill ratio) and orientation (infill pattern). 

Strength, toughness, and geometric accuracy of the manufactured parts depend on various process parameters such as infill ratio, infill pattern, layer thickness, layer height and machine settings [14,15,16,17,18]. Using the optimum process-parameter settings can greatly improve the mechanical strength, surface quality and geometric accuracy by a considerable amount [10,15,19,20,21,22]. Modeling of parts by FDM technique implies also analytical models; some techniques being employed in the simulation models are presented in current work [23,24]. An important role to a reliable model and strength evaluation of the parts manufactured by fused deposition modeling is the proper estimation of bonding between the filaments within a layer (intra-layer bonding) and bonds formed between the filaments of the two successive layers (termed as ‘inter-layer bonding’). The bonding quality among filaments in FDM parts is an important factor in determining mechanical properties of the parts [24,25]. Meso-structure and void density analysis are usually necessary for theoretical calculation of strength and E-modulus of the printed structures. Also, finite element analysis can be used to predict the mechanical behavior of the FDM prototypes [14,26,27,28]. The analysis method proposed by Garg et al. [14] uses an FE method wherein the modeling process is accurately replicating the real inter-layer and intra-layer necking of the filaments during the diffusion of the raster layers throughout the printing process. Three different layer heights and three different raster angles were analyzed by FEM using the ABAQUS platform to simulate the elastic-plastic deformation under a uniaxial tensile load. Experimental studies using fractographic analysis are also performed to validate the results.

The present work presents a novel method to simulate the 3D-printed parts manufactured by FDM technique. The method is based on original G-code of the 3D printer to generate the single filaments. Based on experimental evaluation of the cross-sectional geometry of a printed tensile specimen, the connection between the filaments is determined and also the flattening effect of the filaments can be counted. Uniaxial tensile tests are carried out to investigate the mechanical property of the 3D printed material. Stress calculation is undertaken based on the outside dimensions of the tensile specimen, not taking into account the material/airgap ratio from a cross-sectional area. The resulting E modulus will not correspond to reality, but the tensile strain could serve as a reference to validate the proposed geometrical models and the corresponding numerical simulations. The methodology was applied for specimens with different infill ratios (20%, 40%, 60%, 80 and 100%) and the variation of E-modulus and tensile strength were determined. Specific characteristics of a simulation such as element length effect on the precision and simulation time were also addressed. The paper ends with the conclusions underlying the advantage of the proposed methodology that allows, if the proper connection between filaments is identified, accurate prediction of mechanical behavior of a part only using FE analysis. 

## 2. Materials and Methods

### 2.1. Geometrical Mesostructure Analysis of the of FDM Printed Specimens

After carefully studying the method developed by Garg et al. [14], a disadvantage was identified regarding its real-life applicability; the method does not take into consideration the shell section of the printed part. In real-life application, it is not possible to print parts without the shell section having a low percentage of infill rate, for example, 20%. The analysis method proposed in [14] would not be feasible to determine the true cross-section area of the printed parts because it is a simplified model, applicable only if the infill percentage is high, for example, close to 100%, and does not consider the effect of the shell section on the mechanical behavior of the printed parts. 

**Figure 1:**
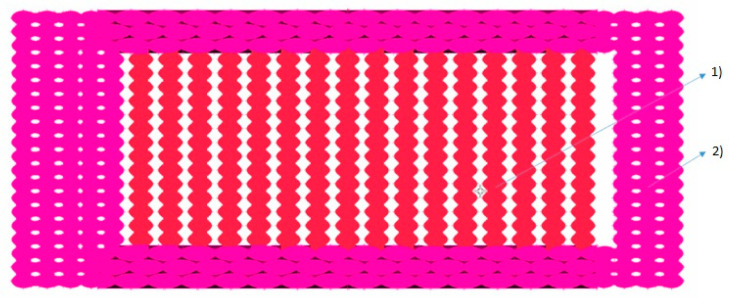
Infill section (**1**) and Shell section (**2**) of a printed ISO_5271A tensile specimen.

Given the lack of specific testing standard for 3D printed materials, selection of dog bone specimens has been undertaken considering their wide use and acceptance for 3D-printed specimens. A cross-section of a 3D printed ISO_527 1A tensile specimen is presented in Figure 1 where both infill and shell areas can be distinguished. Each layer of the specimen is built in the x-y plane by a series of lines parallel to *x*-axis through nozzle movement. The cross-section is lying in the y-z plane, where x, y and z axes are defined by ISO/ASTM 52900:2015.

In order to determine the real area of material in a printed specimen’s cross-section, a new, improved model is necessary. In this study, the efficacy of a geometrical method was analyzed, and designed to be able to build up a very realistic geometrical model of the printed parts, where the material and void ratio is clearly identifiable in a virtual model. This geometrical model should also be suitable to undergo a quick meshing process, where the printed mesostructure will be transformed into finite elements, thus facilitating the prediction of the mechanical behavior of the printed parts using FEM analysis. This method allows us to closely examine the printed parts *Shell* section and *Infill* section as well. The cross-section of the printed tensile specimen is presented in Figure 1.

**Figure 2:**
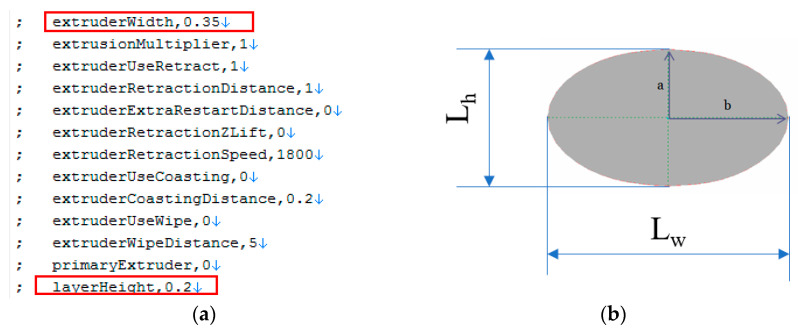
Printed filaments geometry: (**a**) Segment form the printed G-code; (**b**) Theoretical dimensions of a fiber (L_w_ and L_h_ correspond to extruder Width and layer Height).

**Figure 3:**
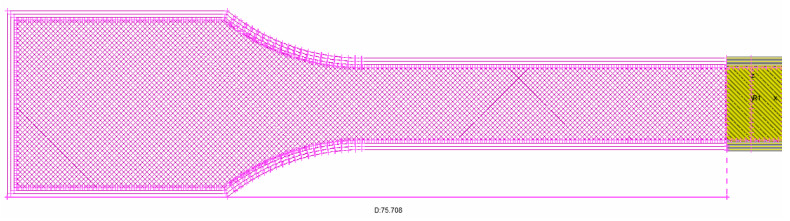
Cross-section distance from the edge of the tensile specimen.

To determine the real cross-sectional area of the specimens with different infill rates, a geometrical method has been employed. In order to create a realistic 3D model of the specimens, G-codes of the printed parts were used to build up the geometrical model. Based on the G-code, which was used for printing the specimens, a geometrical model was constructed in ANSA 17.1.2 (BetaCae, Kato Scholari, Greece). A plug-in script was created in Beta Scripting language, which reads the information from the G-code and transforms the movement of the tool into geometrical lines. The dimensions of the geometrical lines—representing the printed fibers—are identical with the primary layer height of 0.2 mm; the width is considered the extruder width at 0.35mm, as presented in Figure 2, and these dimensions are used to determine the real cross-sectional area of the specimens, using cutting planes transversal to the longitudinal direction of the printed fiber, shown in Figure 3.

The height of the fiber is considered the primary layer height (L_h_ = 2a); the width is considered equal with the extruder width (L_w_ = 2b) values of the printing parameter, according to G-code. The theoretical area of a single filament can be calculated now, using the formula A = πab (a and b from Figure 2). For extracting the area of the cross-section of a printed specimen, a geometric method is used. The virtual (CAD) tensile specimen is constructed in ANSA according to the process described above. Initially five cases of standard ISO_527 1A tensile specimens were studied, where the infill rate ranged from 20% to 100%, the printing direction was kept the same, 0-degree according to the longitudinal axes of the specimen. In all of the five cases the extraction of the cross-section was undertaken in the same place, which is 75.708 mm from the edge of the tensile specimen, as shown in Figure 3.

**Figure 4:**
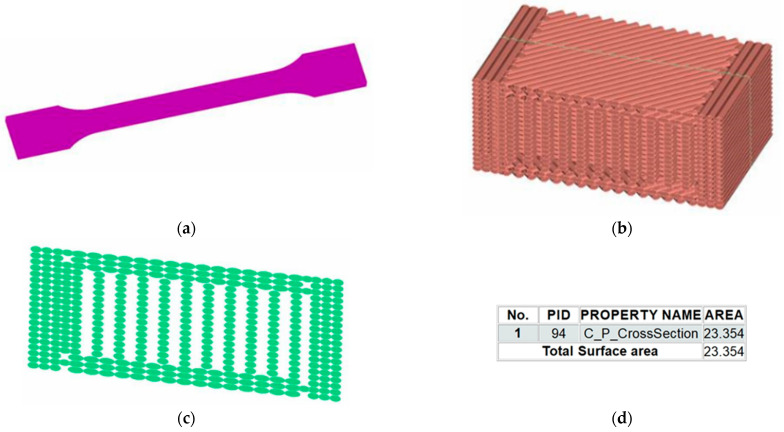
Geometrical modeling and cross-section extraction in ANSA software: (**a**) geometry of the tensile specimen; (**b**) geometrical representation of the filaments; (**c**) cross-section of the geometrical model; (**d**) the result of the area calculation for the cross-section.

The extraction of the cross-section area for the tensile specimens was undertaken in ANSA; the geometry of the tensile specimen is presented in Figure 4a. The geometrical representation of the filaments is shown in Figure 4b. The first step was to define a different property for the face, which was used to intersect the sample; it was named Cutting Plane. The File-Intersect function was used in ANSA to intersect the sample with the Cutting Plane, and the resulting cross-section was assigned a different property named C_P_CrossSection, as represented in Figure 4c. The surface area of the property C_P_CrossSection can be measured using the D.Util function; the result is shown in Figure 4d. The filament representation as a perfect eclipse is a theoretic assumption. 

In reality, the molted material is slightly compacted, and the cross-sections of the filaments are irregular. To verify the area determination method using a CAD model in ANSA, microscopic study of printed specimens is necessary. Five set of tensile specimens were printed using the same G-code’s as in the CAD model construction in ANSA, and the cross-section is analyzed under a microscope to determine the real shape and size of the filaments. 

### 2.2. Estimation of Contact Areas of the Printed Specimens

**Figure 5:**
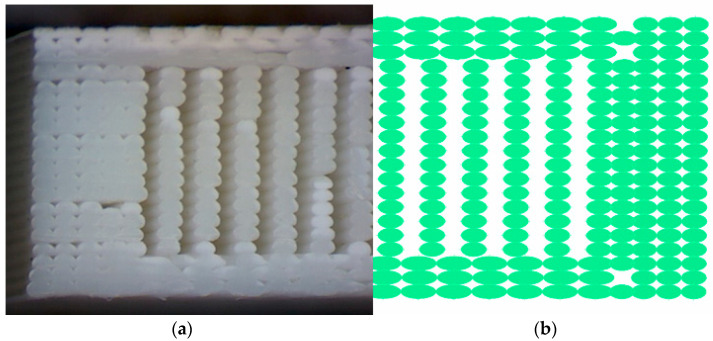
Cross-section comparison of the fiber structure between a printed tensile specimen and the geometrical model in ANSA: (**a**) printed specimen under microscope; (**b**) geometrical model.

The cross-section, meso-structure and infill pattern of the printed specimens was analyzed under a microscope, with 50× magnifying factor, to determine the real shape and structure of the fibers. The result of the cross-section extraction from the geometrical model was compared to the microscopic pictures of the printed specimens. Because the extruded filament is in semi-molten state, both its top and bottom would flatten slightly when deposited onto the previous layer, as indicated in the microscopic photo Figure 5a. Correlation of the geometrical area is necessary, because the cross-section of the fibers are represented as a perfect ellipsoid in the geometrical model; however, in reality, the cross-section of the fibers has a bigger contact patch, due to the fact that their cross-section is not perfectly ellipsoid shaped, as presented in Figure 5b, and pointed out in [13,23].

**Figure 6:**
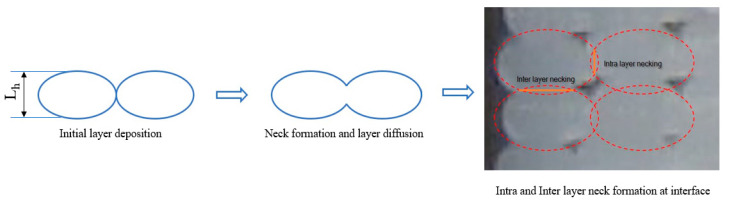
Necking formation between adjacent raster layers.

Therefore, a modified calculation method is required when considering the flattening effect that can be measured experimentally. This flattening effect leads to the formation of a bigger contact area between the filaments, known as intra- and inter-layer necking, as presented in Figure 6.

**Figure 7:**
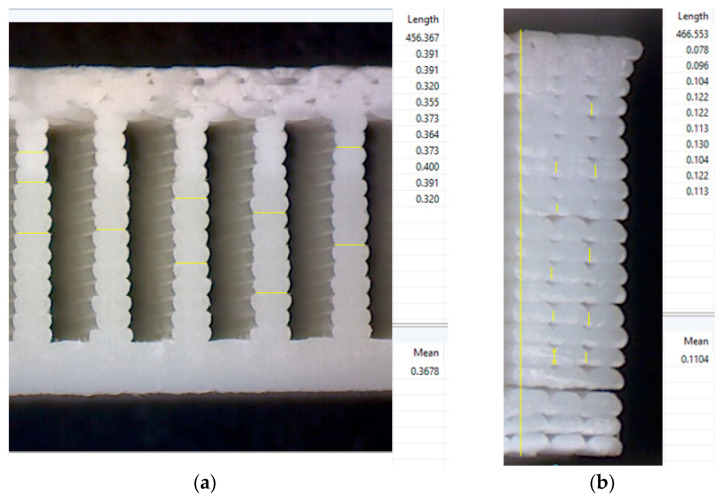
Determination of the contact area between the filaments (**a**) inter layer necking; (**b**) intra layer necking.

In the case of using only the printing direction or infill pattern to build up a part or a sample, determination of the real cross-section area is fairly easy, considering that the areas of inter-layer and intra-layer necking can be determined experimentally by optical measurements. The void and material ratio are practically an adjusted area of the printed filament layer multiplied with the number of layers. In the geometrical model, the cross-section of the fibers will be considered perfectly ellipsoidal, and the relationship between the printing parameters and settings can be established. For example, if the relationship between the printing parameters (primary layer height, extruder width) and the proportion of the flattening effect is found, the real area of the fibers can be accurately predicted, and the information implemented in the script, which calculates the fiber cross-section area. A scale factor has to be established to create the correct assumption of the cross-section area of the individual fiber, which will be used to correct the geometrical model area calculation errors. The scale factor is determined by optical analysis of the cross-sections of different specimens under microscope. To determine the real size of the contact patch between the layers (inter-layer necking and intra-layer necking), according to infill rate and infill pattern, microscopic pictures were analyzed with Digimizer software (MedCalc Software Ltd., Ostend, Belgium), where the length of the contact areas, both inter-layer and intra-layer necking was measured, as presented in Figure 7.

**Figure 8:**
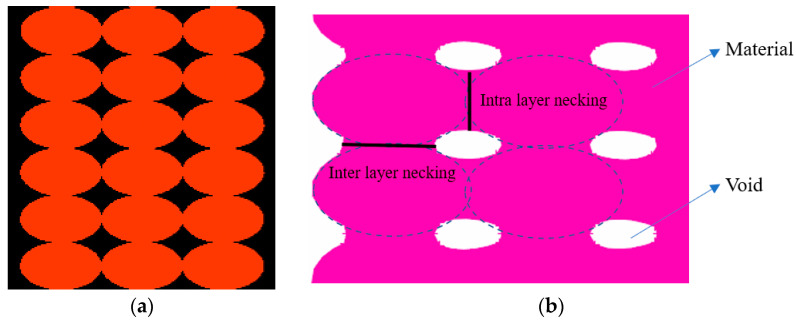
Representation of the filament: (**a**) initial geometry; (**b**) adjusted to represent the intra and inter layer necking.

Taking into account the values of the horizontal (inter-layer necking) and vertical overlapping (intra-layer necking) determined experimentally, an updated geometrical model was created wherein the filaments are still represented as a perfect ellipsoid, but the contact area between the layers was modified to fit the measured intra- and inter-layer necking, as shown in the Figure 8. 

It can be observed that the inter-layer necking is close to the extruder width (extruder with 0.35 mm, inter layer necking ~0.36 mm) and the intra-layer necking is about half of the primary layer height (layer height 0.2 mm, intra-layer necking ~0.11 mm). Taking into account these observations, the geometric model was updated in order to better approximate the geometric model to reality, thus facilitating a more realistic cross-section area determination. Furthermore, if the filament area is accurately determined for different printing parameters, the air-gap material ratio can be found to determine the density of the printed specimens. To validate the method, real-life tensile tests are necessary, where the results of the tensile test, especially tensile strain will be compared to the result of numeric simulation, therefore validating the geometrical model. Another important fact should be taken into consideration, i.e., when determining the area of the cross-section by geometric method in ANSA, the area of the cross-section is dependent on the settings in ANSA, especially “Perimeter length” and “Resolution tolerance”. Perimeter length defines the node density on the edges. A high node density (shorter distance between the nodes) allows a finer mesh generation, which better approximates the mesh to the edges of the geometry, thus minimizing the loss of material (cross-section area in our case). Resolution tolerance displays the geometrical details in high or lower resolution. A fine tolerance displays all the features in high detail, and also allows a low perimeter length, but uses a lot of computing and graphic resources, which can make a model difficult to work with.

### 2.3. Finite Element Analysis of FDM 3D Printed Specimen

Finite element analysis is conducted on the geometrical models, described in Section 2.1. In order to validate the geometrical model proposed, the result of the finite element analysis and experimental data should match. To verify the cross-section of the specimens, numerical simulation will be performed on the geometrical models where the resultant tensile strain will be compared with the experimentally obtained values. If they are comparable, the area of the cross-section is correct and the FEA1 and FEA2 tensile moduli are considered correct. After the tensile moduli are determined for different infill rates, they can be used for FE simulations utilizing a homogenous mesh.

To obtain accurate results in Finite Element simulations the model creation part is especially important. The model should be built up as precisely as it can be, and all the boundary conditions should be represented and applied as realistically as possible. Choosing the right representation of the modeling approach, element type, element length and quality file is also crucial. Conventional Finite Element modeling techniques are not suitable for analyzing 3D-printed parts; due to the air gaps inside the parts, they cannot be considered either simple shell or volume parts. The modeling method used in this study is utilizing the experience of Garg et al. [14], where the authors used a microstructure modeling approach, wherein each filament line is modeled with Tetra elements and they are connected according to previously established intra-layer and inter-layer necking. As pointed out earlier in this document, this approach presents some limitations in terms of applicability in case of functional parts, where the printed parts are more complex and are built up with a shell section around the infill area. In order to eliminate this problem, the shell section should also be accurately modeled, and the modeling time should be reduced and if possible automatized. The method proposed desires to meet this requirement, where the modeling can be at least partially automatized in order to reduce modeling time, and capability to build up complex models with shell section ca be included. 

The process used in this study starts with the initial geometrical model, built up in a conventional design tool. The CAD model will be processed in a “slicer tool”, wherein the printing parameters are defined. The result of this process will be a G-code, which is readable by 3D printers. The G-code contains all the information that the 3D printer requires, such as temperature of the extruder head, layer height and width, printing speed and, most importantly, all the coordinates of the extruder heads movements. This coordinates and printer settings can be used to create a very realistic micro-structure model, which is suitable for later Finite Element analysis. 

**Figure 9:**
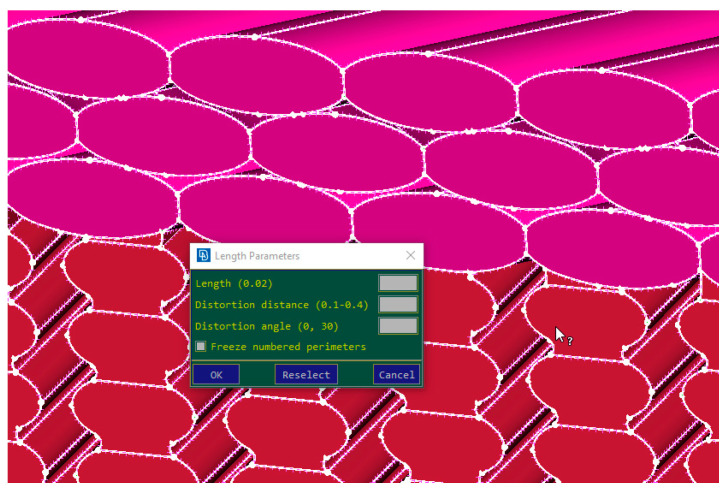
Setting the Perimeter Length to a value that allows a precise representation of the layers.

The process can be described as follows: the geometrical models were established in ANSA 19.1.2 using a script that translates the movement of the extruder head based on the printed G-code and transforms the movements of the extruder of the 3D printer to geometrical lines. The geometrical lines are then used to extrude an ellipsoid section of the infill along the lines laid down in the previous step. The shape of the individual layers is determined by the primary setting of the printing parameters, for example, primary layer height, extruder diameter, etc., which the script reads from the G-code and creates the primary ellipsoid accordingly. The ellipsoid section is adjusted with the overlapping necking areas, (inter-layer and intra-layer necking) in order to adjust the perfect geometrical model to a more realistic model, wherein the printed layers do not have a regular ellipsoid shape, rather a deformed quad-like shape. In order to extract the cross-section area of the tensile specimens, the created geometry has to be meshed. In the software ANSA, there is a possibility to obtain information about surface areas, but the surface representation in term of accuracy is connected to “perimeter length”. Perimeter length finally defines the length of the mesh created on the surfaces. To extract the real cross-section area as precisely as possible, a very low perimeter length was used: 0.02, as shown in Figure 9.

**Figure 10:**
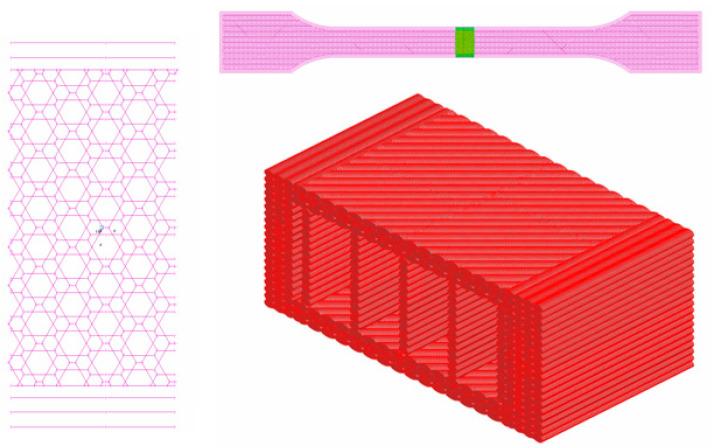
FEM representation of the filaments. (Segment from a standard tensile test specimen).

After the determination of the cross-section area of the tensile specimen, Finite Element analysis was conducted on a portion of the parallel part of the tensile specimen. In order to decrease the simulation time and to fit the processing power of a generic workstations, only a segment of the tensile specimen was analyzed. The length of the segment is 5.43 mm, elected randomly with the consideration to have at least four repetitive elements in case of complex infill patterns such as Honeycomb or Triangle, as shown in Figure 10.

Several simulations were conducted in order to determine the optimal modeling procedure. The factors that influence the result are element type, element order, element length and quality of the elements. The first five sets of models (with infill rate from 20%, 40%, 60%, 80%, 100%, direction: 0° according to the longitudinal axis of the sample) were build up using Tetra, first order elements, with a generic element length of 0.05 mm. The models were loaded with 160 N on one end, and fixed on the other with a rigid body *RBE2* element connected at the center of gravity to a single point of constrain (*SPC*), which forbids all movements and rotations.

**Figure 11:**
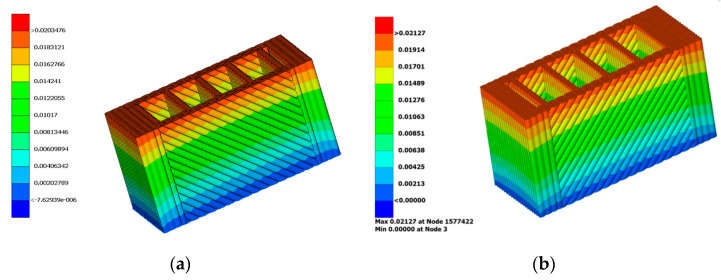
Result of simulation in: (**a**) Epilysis and in (**b**) Nastran.

Linear static simulations, assuming isotropic material, were conducted in Epilysis, which is a solver provided by BETA CAE, and it is integrated in the pre-processing tool ANSA. Epilysis is using a Nastran source code, and the results are comparable with the results obtained in Nastran. In fact, the first model was simulated in Nastran and in Epilysis too, and the output was compared, resulting in displacement difference of *4.3%* (from 0.0203476 to 0.02127), as presented in the Figure 11.

Although the Nastran delivers a more precise result than Epilysis, the further simulations were performed in Epilysis because of the availability of licenses, and because the average result of the simulations performed with Epilysis are off by 5.2%, (compared to experimental results), which can be considered acceptable. The post-processing of the data was undertaken in Animator_v2.0.8 for the result of Nastran, and in Metapost 19.1.2 for the results of the simulations performed in Epilysis. The two post-processing softwares were compared also, and no significant difference was found in reading the displacements and stresses from the resulted.op2 files. 

A model with Tetra 2nd order element was built up and simulated using the exact same boundary conditions as the 1st order model. In case of Tetra 2nd order model, the total number of elements are the same, but the number of nodes are increased from 373,777 to 2,738,200 (in case of 100% infill) due to the introduction of the middle nodes on each element edge. The results are showing a highest deformation in case of 2nd order element, for example, in the case of 40% infill rate where the experimentally measured displacement was 0.00352 mm, the simulation result was 0.00369105 mm, which represents a 4.63% increase in displacement. After analyzing the results, we can notice, that the simulation model built up using tetra 1st order elements is always showing a deformation less than the experimentally obtained values, which is expected due to the fact that Tetra 1st order elements are stiffer than Tetra 2nd order elements. 

As expected, the simulation time was gradually increasing with the complexity and size of the models varying from 12 min for model with the lowest infill rate 20%, up to 45 min for the highest infill rate 100%. With the increase in infill rate from 20%- to 100%, the models increased from (element length 0.05) 3,740,121 tetra element to 11,940,382 elements, which can be expressed as a 219.25% increase. In this case, a regular personal computer is unable to process the amount of information, therefore, professional calculation/simulation servers are recommended. 

**Figure 12:**
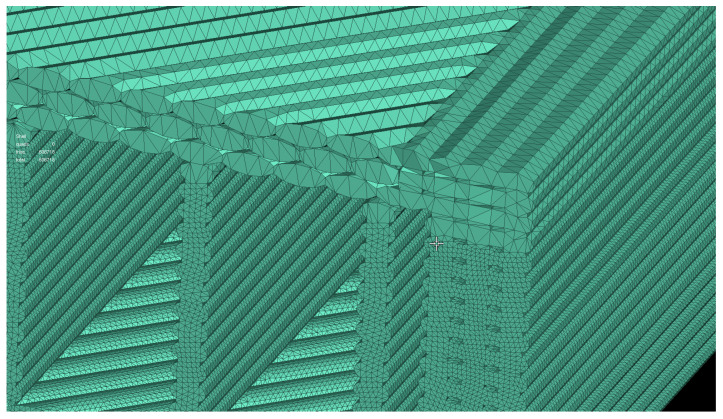
Influence of the higher target length on the representation of the individual filaments.

In order to analyze the effect of the element length on the precision and simulation time, another model setup was tested. The element length was doubled from 0.05 to 0.1 mm. The simulation time of the models with 0.1 mm element length varied between minimum 1 min for 20% infill rate up to 8 min for the more complex model of 100% infill rate. Comparing the two models in terms of the computing time: using a larger element length (0.1 mm) reduced the time with a factor of 5.6, while delivering an average precision of results with an error of 7.5% compared to the experimental results and 2.3% compared to finer mesh (0.05). The differences are caused by the loss of details in geometry due to the representation of the layers in a simplified way, as shown in Figure 12.

After reviewing the effects of the FE model build parameters, the simulation and post-processing tools, it can be concluded that the “Best Practice” for our investigation concerning FE analysis is as follows: the Tetra 1st order element model, with a global element length of 0.1 mm. The pre-processor will remain Ansa, solver: Epilysis and post-processing tool: Metapost. This approach to modeling seems to be a well-balanced solution between accuracy of results, computing time and the need for processing power. Another conclusion can be drawn from analyzing the result of the simulations, i.e., that using a bigger element length greatly reduces the computation time, while delivering the results with an acceptable precision.

### 2.4. Tensile Test 

The geometric models of 3D-printed specimens were realized according to ISO 527-2-2012 (International standard, plastics Determination of tensile properties Part 2: Test conditions for molding and extrusion plastics). Uniaxial tensile tests are carried out to investigate the mechanical property of the 3D-printed material. The specimens are tested with a universal testing machine type INSTRON 3366, 10 kN capacity. The loading speed of this machine is 1 mm/min and the test stops once the specimens are broken. A uniaxial extensometer was used to measure the tensile strain. For each infill rate, a number of five specimens were tested. The material used for specimen’s preparation was acrylonitrile butadiene styrene ABS filament (Plasty Mladeč, Czech Republic). Its mechanical properties, according to the producer, are as follows: tensile modulus E_f_ = 2140 MPa, tensile stress σ_f_ = 43 MPa and tensile strain ɛ_f_ = 2.7%. 

Stress calculation is undertaken for all infill rates based on the cross-sectional dimensions of the tensile specimen (4 mm thickness, 10 mm width; not taking in account of the material/airgap ratio, results in a cross-section area of 40 mm^2^). The resulting tensile stress will not correspond to reality, but the tensile strain could serve as a reference to validate the geometric model with numeric simulations. If the experimental tensile strain is comparable to the one obtained by FE simulation, the area of the cross-section is validated. 

A tensile test simulation was conducted on the FE model using Epilysis solver. Previously, the same tensile specimen was tested experimentally. The same boundary conditions were applied to the FE model as for the real tensile specimen during the tensile test. The objective of the simulation was to find out if the elongation of the FE model corresponds with the experimental results. The results can be summarized by Table 1 where the experimentally measured strain is compared to the results of the FEM simulations performed with a finer (0.05 mm) and a bigger element length (0.01 mm) and the deviation between them is expressed as percentage. The results are selected from the elastic domain of the material at 160 N load.

Ten simulations were conducted to determine the effect of the element length on the precision of the result. The basis of comparison is the displacement obtained by real experiment (tensile test) in relation to the FE results using two different element lengths, a finer mesh 0.05 and course mesh of 0.1 mm. The results showed a range of minimum 3.13% and maximum 8.05% difference in the case of FE model meshed with 0.05 mm element length (FEA1) compared to the experimental result (EXP). The FE model meshed with 0.1 mm element length (FEA2) delivers results with a slightly higher error, where the range is minimum 6.42% and maximum is 8.66%. Comparing the two FE models, the range is between 0.55% and 3.09% difference, which is considered a good result factoring in the savings in terms of processing power and simulation time, as mentioned in the previous paragraph. A convergence is noticeable when comparing the two FE models (Table 1—Relative Dev. FEA1- FEA2), with the increasing of the infill rate. 

The obtained results are consistent with the measured data that proved that our estimations of contact areas and filament shape of the printed specimens and the proposed finite element models are accurate, and it can be successfully further employed. 

## 3. Results and Discussions

**Figure 13:**
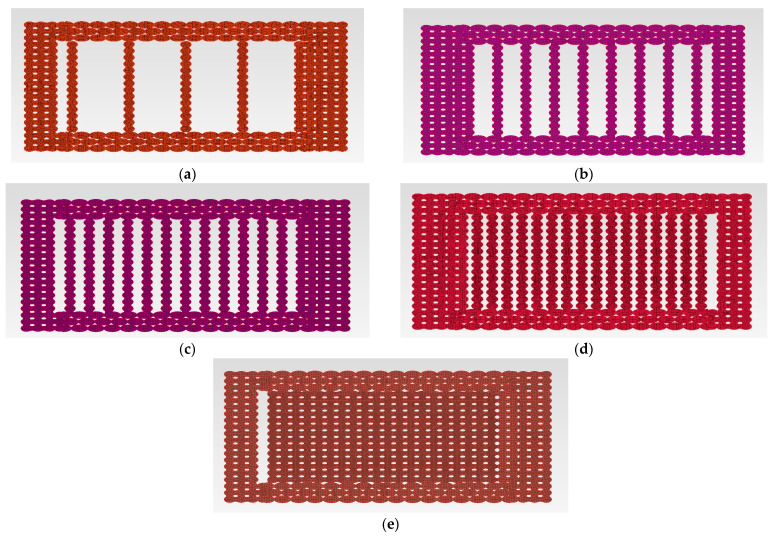
Cross-sections patterns according to infill rate: (**a**) 20%; (**b**) 40%; (**c**) 60%; (**d**) 80%; (**e**) 100%.

In Figure 13, the cross-section of the tensile specimens is presented (as they are extracted from the geometrical model from ANSA) from 20% to 100% infill rate.

The first set of cross-section values are representing the areas of the samples printed with different infill rates, determined with a perimeter length (PL) of 0.05 mm. The value 0.05 mm is the mesh length used for FE simulation (Perimeter length = Mesh length) to validate the geometrical model based on tensile strain comparison. As previously presented, two different meshing lengths have been used to simulate the specimens, a finer mesh: 0.05 mm (FEA1) and a coarser mesh: 0.1 mm (FEA2).

In the case of FEA1 and FEA2, the area of the cross-section was extracted from the geometrical model from ANSA, as presented in Table 2, and introduced into the testing machine software allowing for a more accurate calculation of the tensile stress.

The increase in the cross-section area FEA1 and FEA2 is, on average, 13%, so the 20% increase in “Infill rate” does not result in a similar increase step in cross-section. Examining the quantity of raw material (filament length and material weight) added to specimens with different infill rate (Table 3), values delivered by the 3D-printer software, a decrease from 20% to 13% can be observed. A possible explanation is that a change in the infill rate determines not only an increase in the quantity of the deposited filament, but it is also constrained by the disposal (pattern) of the filaments according to the part geometry.

**Figure 14:**
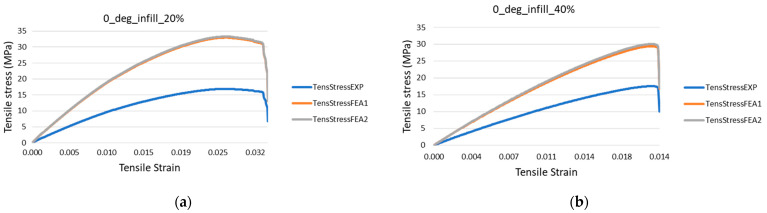

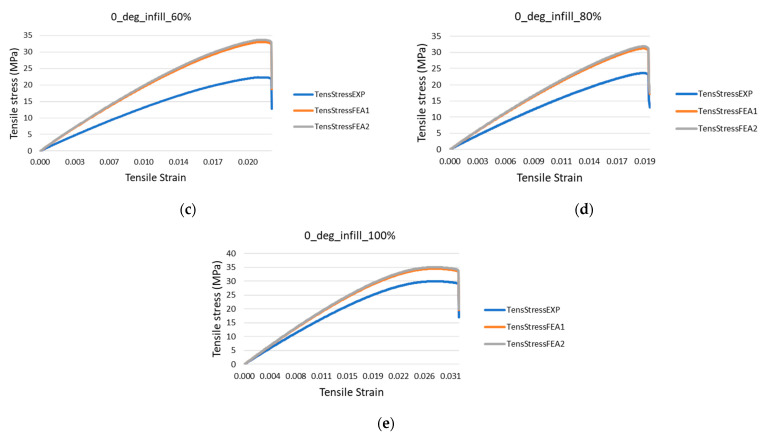
Stress-strain curves recalculated based on numerically estimated cross-sectional areas specimens with the infill rates: (**a**) 20%; (**b**) 40%; (**c**) 60%; (**d**) 80%; (**e**) 100%.

Comparative strain-stress curves for all analyzed infill rates are presented in Figure 14. The initial experimental curve (EXP) is based on a constant cross-sectional area of 40 mm^2^ given by the outside dimensions of the specimens. The other two curves (FEA1 & FEA2) presented in Figure 14 are recalculated experimental strain-stress curves considering numerically estimated cross-sectional areas. It can be observed that utilizing the full cross-section of the 3D-printed specimen, even for a 100% infill rate, will not deliver a result within an expectable error range. 

In order to predict the tensile modulus of the 3D-printed specimen with different infill rate, the cross-section extracted from the geometric model was reintroduced into the testing machine software and the results were recalculated according to the two set of values. 

In Table 4, the result of the tensile modulus is presented where: E modulus EXP is representing the tensile modulus of the specimen—experimentally determined—utilizing the full cross-section of the sample; E modulus FEA1 and FEA2 are representing the recalculated tensile moduli of the specimens utilizing the area extracted from the geometric model employing the finer mesh (0.05 mm) and the course mesh (0.1 mm) definition, respectively.

**Figure 15:**
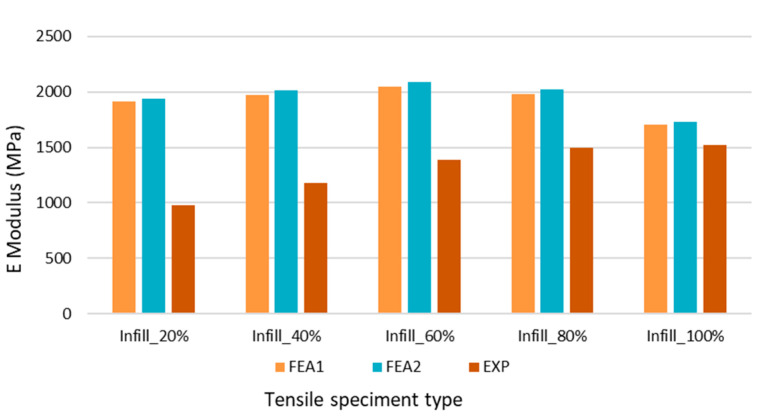
E-moduli value for different infill rates recalculated based on numerically estimated cross-sectional areas.

The influences of the perimeter length on the area of the cross-section (which translates to the values of the E modulus) are presented in Figure 15. It can be observed that there is an inverse correlation between the numerically and experimentally determined E modulus in relation to the infill rate. In case of a low infill rate of 20%, the difference between the E modulus EXP and E Modulus FEA1 is 53%, while for an infill rate of 100%, the difference between E modulus EXP and FEA1 is reduced to a still significant 36%. It can be concluded that for tensile modulus, utilizing the full cross-section of the 3D printed specimen, even for a 100% infill rate, will not deliver a result within an expectable error range.

Comparing the deviation between E-modulus FEA1 and FEA2, we can observe an average of 1.72% difference, which is coming from the mesh size difference. As expected, for higher infill rates, the values of FEA1 and FEA2 are converging to the experimentally obtained (EXP) values, but contrary to our initial expectation, not in a proportional manner. The results are clearly showing that the increase in 20% in infill rate does not directly translate to a 20% change in E modulus. In the case of the experimental results, the difference from EXP Infill_20% to EXP Infill_40% is a 19.9% step, but this shrinks to 2.1% when comparing EXP Infill_80% to EXP Infill_100%. Notably, the values of FEA1 and FEA2 are increasing, up to Infill_60%, then slightly decreasing to Infill_100%.

**Figure 16:**
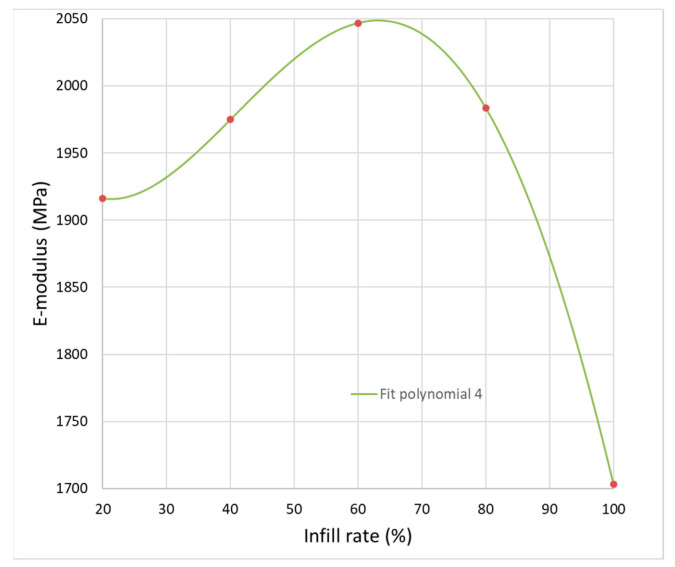
Polynomial fit of the infill rate vs. E-modulus of tensile specimens.

Variation of E-modulus with respect to the infill rate is depicted fin Figure 16. A quadratic polynomial fit delivers the best results. 

Analyzing the obtained equations of the fitting curve:

E-modulus/MPa = 2085 − 18.6 Infill rate/% + 0.6307 Infill rate^2^/%^2^ − 0.006569 Infill rate^3^/%^3^ + 1.74 × 10^−5^ Infill rate^4^/%^4^ (1)

it can be observed that the initial term (2085) is very close to the value of measured E-modulus of the filament (E_f_ = 2140 MPa), and has a slight increase up to 60% infill rate and then a decrease.

In order to fully understand and explain this behavior, a closer analysis on the cross-sections is necessary (Figure 13, Table 2 and Table 3). Inconsistencies can be observed in orienting the infill pattern into the shell section of the printed part, where the shell section has a wall that consists of three rows of filament on one side and five rows of filament on the other, shown in Figure 11. This effect can be especially important at high infill rate (Infill 80%, 100%) and can influence the way the infill is connected to the shell part of the specimen, which can result in different bonding forces between filaments, on one hand, and inner filaments and outer shell, on the other hand. A stiffness difference between the left and the right sides can be also produced. Our results indicate that besides strength reported in [24], the E-modulus of the FDM specimen is not only dependent on the infill rates, but an important contribution is made by intra-layer bonding, inter-layer bonding and neck growth between filaments. As long as the printer is not respecting a symmetrical disposal of the rows of filaments and outer shell construction correlated with the infill rate such behavior can occur and can be estimated only by applying a methodology as those presented in this paper.

## 4. Conclusions

In this paper 3D-printed tensile specimens were analyzed in order to understand the effect of different infill rates on the mechanical behavior. A novel approach to analyze 3D-printed FDM models was presented, which is utilizing a geometrical model, constructed based on the printer generated G-code, which facilitates Finite Element analysis. The issue of cross-section determination was addressed, and a geometrical model was developed to investigate the “air gap—material ratio” problem. A case study was conducted on finite element models in order to establish the best modeling method for optimal balance between accuracy and simulation running time. The tensile strain resulted when the two cases of simulations (FEA1 and FEA2) were compared to experimental result, which confirmed that the area of cross-section extracted from the geometric model is predicted with good accuracy.

In order to determine the E moduli of different infill rates, the cross-sections extracted from the geometric model were reintroduced into the tensile testing machine. After conducting twelve simulations, we can establish that the simulation method proposed in this study is a viable option to predict the behavior of 3D printed parts even before they are being printed, only by running an analysis on the G-code generated by the “slicer” tool. The obtained E moduli for different infill rates can be used for FE simulation where the microstructure no longer has to be modeled; a simple volume mesh should be enough because the E modulus also contains the correct airgap-material ratio. 

The findings presented in this paper allow the following overall conclusions to be drawn:

- The proposed approach for constructing a complex finite element model based on the printer generated G-code is a reliable methodology to predict the behavior of the FDM printed parts but adjustments to represent the intra- and inter-layer necking are necessary for accurate results.
- Cross-sectional area of a tensile specimen extracted from the numerical model is predicted with good accuracy and allows estimation of strain-stress curves and E-moduli closer to reality.
- For higher infill rates the values of tensile stress and E-modulus of the specimens are converging to the experimentally obtained values, but not in a proportional manner.
- The results are clearly showing that the increase in the infill rate does not directly translate to a corresponding change in E modulus, disposal of the rows of filaments influencing the bonding forces between them and the outer shell.

The main purpose of this modeling method is to being able to model complex 3D-printed parts with variable infill rate and tunable stiffness. If the right method to connect the single filaments is established for a specific printer (accounting printing speed, resolution, nozzle diameter, temperature, material behavior during deposition, etc.), and tested to provide a reliable result, the mechanical properties of the 3D specimens can be predicted without physical tensile test, which will allow the designers to print out the parts with variable infill rate only after the FE result are suitable for their needs, saving considerable materials and time. If the computation power allows, we consider that the methodology can be extended to complex parts. Having information about the mechanical behavior of the parts before the actual printing could be important in the design strategy of functional prints or in applications with a reduced number of filaments such as 3D-printed houses.

## Figures and Tables

**Table 1:** Comparison of the displacements from experimental tensile test (EXP) to simulation results with two different element lengths (FEA1 & FEA2).

Infill Rate (%)	Strain EXP (mm/mm)	Strain FEA1 (mm/mm)	Strain FEA2 (mm/mm)	Relative Dev. FEA1-EXP (%)	Relative Dev. FEA2-EXP (%)	Relative Dev. FEA1-FEA2 (%)
20%	0.004040	0.003917	0.003796	3.14	6.43	3.09
40%	0.003520	0.003300	0.003250	6.65	8.31	1.53
60%	0.003040	0.002907	0.002866	4.57	6.06	1.40
80%	0.002740	0.002584	0.002561	6.04	6.97	0.87
100%	0.002510	0.002323	0.002310	8.06	8.66	0.56

**Table 2:** Estimated cross-sectional areas according to infill rate and mesh size.

Infill Rate	Cross-Sectional Area of the Specimen (mm^2^)	
	FEA1	FEA2
20%	20.50	20.30
40%	23.85	23.35
60%	27.05	26.52
80%	30.23	29.66
100%	34.72	34.19

**Table 3:** Filament length and specimen weight variation with the infill rate.

Infill Rate Change	Filament Length (%)	Material Weight (%)
20–40%	19.07	18.99
40–60%	16.04	15.96
60–80%	13.83	13.88
80–100%	13.57	13.57

**Table 4:** Tensile modulus of the printed specimens with geometrically determined cross-section.

Infill Rate	E-Modulus (MPa)		
	FEA1	FEA2	EXP
20%	1916.0	1934.9	982.0
40%	1974.8	2017.1	1177.5
60%	2046.7	2087.7	1384.1
80%	1983.4	2021.5	1498.9
100%	1703.4	1729.8	1521.8

## Data Availability

Not applicable.
